# Idiopathic segmental infarction of the omentum mimicking acute appendicitis: A case report

**DOI:** 10.1016/j.ijscr.2019.03.050

**Published:** 2019-04-06

**Authors:** Rahul Gupta, Waad Farhat, Houssem Ammar, Mohamed Azzaza, Sami Lagha, Yesser ben Cheikh, Mohamed Ben Mabrouk, Ali Ben Ali

**Affiliations:** a Department of Gastrointestinal Surgery, Synergy Institute of Medical Sciences, Dehradun, India; b Department of General and Digestive Surgery, Sahloul Hospital, University of Medicine of Sousse, University of Sousse, Tunisia; c Department of Radiology, Sahloul Hospital, University of Medicine of Sousse, University of Sousse, Tunisia

**Keywords:** Omentum, Laparoscopy, Infarction, Appendicitis

## Abstract

**Introduction:**

Omentum is a well vascularized organ. Omentum infarction is uncommon. Most of the cases are secondary due to torsion, intra-abdominal infections and vascular thrombosis. Primary idiopathic segmental omental infarction is rare.

**Presentation of case:**

A 26-year-old male presented with acute onset right iliac fossa pain mimicking acute appendicitis. On radiological imaging, early acute appendicitis was suspected. On diagnostic laparoscopy, appendix appeared normal and there was idiopathic infarction of a part of the greater omentum lying close to the cecum. Patient underwent laparoscopic appendectomy with excision of the diseased segment of the omentum and had uneventful recovery.

**Conclusion:**

Idiopathic omental infarction should be included in the differential diagnoses while treating patient with acute abdomen.

## Highlights

• Omentum infarction occurs due to torsion, infections and vascular thrombosis.
• Primary idiopathic segmental omental infarction is rare.
• Clinically it is difficult to differentiate from acute appendicitis.
• Idiopathic omental infarction should be included in the differential diagnoses while treating patient with acute abdomen.

## 1 Introduction

Pain in right iliac fossa is a common clinical problem. Most of these cases are due to acute appendicitis, renal stones, tubo-ovarian diseases or acute cholecystitis. Omentum is often secondarily involved in the inflammatory process. Primary omental diseases leading to abdominal pain are rare. Moreover, preoperative diagnosis of omental disease in the absence of intestinal or solid organ involvement is challenging and requires high index of suspicion.

Primary idiopathic omental infarction is a rare disorder to affect young adults. It is diagnosed intraoperatively in most of the cases. If diagnosed on radiology, then conservative treatment can be offered [1]. We present an unusual case of segmental omental infarction mimicking acute appendicitis that was diagnosed intraoperatively and treated successfully by segmental resection of the diseased omentum. This case has been reported in line with the SCARE criteria [2].

## 2 Case description

**Fig. 1:**
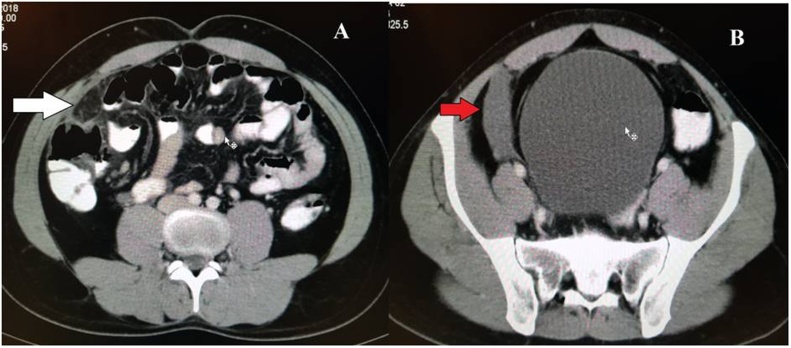
Contrast enhanced computed tomography showing omental fat stranding (white arrow) and free fluid in the right iliac fossa (red arrow).

A 26-year-old male presented with complaints of acute onset right iliac fossa pain associated with nausea and vomiting for 4 days. On clinical examination, right iliac fossa tenderness was present. Blood investigations were within normal limits. Ultrasonography revealed presence of mild free fluid in the right iliac fossa. Appendix could not be visualized. On contrast enhanced computed tomography (CECT) abdomen and pelvis, there was presence of free fluid in the right iliac fossa with thickening of the right conal fascia and omental fat stranding (Fig. 1). The lumen of the appendix was patent and the tip of the appendix appeared to be thickened. Based on these findings, clinical diagnosis of acute appendicitis was made and patient was planned for laparoscopic appendectomy.

**Fig. 2:**
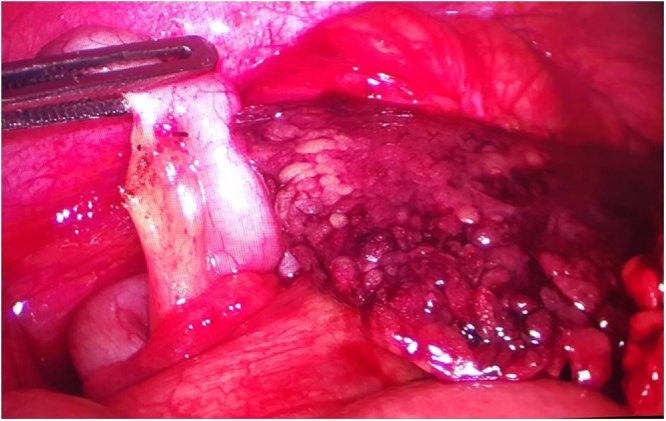
Intraoperative photograph showing the infarcted omentum and normal appendix.

On laparoscopy, about 200 ml of hemorrhagic fluid was present in the right iliac fossa and pelvis. A segment of the omentum adjoining the cecum appeared dusky, congested and partially infarcted while the rest of the omentum was normal in appearance (Fig. 2). Grossly, appendix, cecum and terminal ileum appeared normal (Fig. 2). Laparoscopic appendectomy with excision of the diseased part of the omentum was performed. Postoperative recovery was uneventful with two days of hospital stay. On histopathology, appendix was normal with lymphoid hyperplasia while omental specimen showed areas of congestion, hemorrhage and inflammation.

## 3 Discussion

Omental infarction is uncommonly encountered in clinical practice due to its rich vascularity. However, if it occurs, then it can be either primary or secondary [3]. In most of the cases it is secondary to an identifiable cause such as strangulated inguinal or ventral hernia, vascular thrombosis, neoplasms and inflammatory conditions [4]. Primary omental infarction is rare with about 400 cases reported in literature [5]. Obesity is an important risk factor for developing omental infarction. It can be segmental or involve the whole omentum. The right side of the omentum is the most frequently affected part of the omentum as per in the present case [4,5].The probable reason for the right-side predominance is that the omentum on the right side has longer length and greater mobility compared to the left side [5].

The classical presentation of primary omental infarction is abdominal pain predominantly on the right side with presence of local tenderness. Clinically it is difficult to differentiate from other right sided inflammatory disorders such as acute appendicitis. On ultrasonography, it appears as an echogenic mass-like lesion [6]. However, this finding may not be present in every case. But, other differential diagnoses such as acute appendicitis, acute cholecystitis and ectopic pregnancy can be ruled out. CECT of the abdomen is the imaging modality of choice to diagnose omental infarction [4]. In case of omental infarction, there is extensive fat stranding in the area of infarction that is disproportionately greater than the adjoining bowel wall thickening [4]. In some cases, the infarcted omentum can appear as a large, cake-like, high attenuation fatty lesion adjacent to the bowel [4].

Traditionally, primary idiopathic omental infarction was managed by surgical excision. But with increasing number of cases being diagnosed on radiological imaging, it has been found to be a self-limiting disease and can be treated with medical therapy in most cases [1,6]. However, there can be readmission for recurrent or ongoing pain in up to 25% cases [6]. Moreover, some patients on medical therapy may develop omental abscess requiring surgery [7,8]. Laparoscopic excision is the procedure of choice in case of failure of medical management and in cases where upfront surgery is performed due to diagnostic dilemma as seen in our case.

## 4 Conclusion

Idiopathic omental infarction is a rare cause of abdominal pain in young population. It should be included in the differential diagnoses while treating patient with acute abdomen. Conservative treatment can be offered if diagnosed on radiology.

## Conflict of interest

The authors declare that they have no conflict of interest.

## Funding

This study has not received any funding.

## Ethical approval

The study was approved by Ethics Committee.

## Consent

Written informed consent was obtained from the patient.

## Authors’ contributions

Study concept or design – MBM, HA.

Data collection – HA, WF, RG.

Data interpretation – MBM, YB, EH.

Literature review – WF, SL, MM, ABA.

Drafting of the paper – HA, FH, SL.

Editing of the paper – MBM, YB, AM.

## Registration of research studies

As this was a case report and not a clinical trial, this study does not require registration.

## Guarantor

Mohamed ben mabrouk.

Rahul Gupta.

## Provenance and peer review

Not commissioned, externally peer-reviewed.
